# Antinociceptive Effect of a p-Cymene/β-Cyclodextrin Inclusion Complex in a Murine Cancer Pain Model: Characterization Aided through a Docking Study

**DOI:** 10.3390/molecules28114465

**Published:** 2023-05-31

**Authors:** Wagner B. R. Santos, Lícia T. S. Pina, Marlange A. de Oliveira, Lucas A. B. O. Santos, Marcus V. A. Batista, Gabriela G. G. Trindade, Marcelo C. Duarte, Jackson R. G. S. Almeida, Lucindo J. Quintans-Júnior, Jullyana S. S. Quintans, Mairim R. Serafini, Henrique D. M. Coutinho, Grażyna Kowalska, Tomasz Baj, Radosław Kowalski, Adriana G. Guimarães

**Affiliations:** 1 Departament of Pharmacy, Federal University of Sergipe, São Cristóvão 49100-000, SE, Brazil; wagner-b-r@hotmail.com (W.B.R.S.);; 2 Departament of Physiology, Federal University of Sergipe, São Cristóvão 49100-000, SE, Brazil; 3 Departament of Biology, Federal University of Sergipe, São Cristóvão 49100-000, SE, Brazil; 4 Department of Pharmacy, Federal University of Vale do São Francisco, Juazeiro 48902-300, BA, Brazil; 5 Department of Biological Chemistry (DBQ), Regional University of Cariri (URCA), Pimenta, Crato 63105-000, CE, Brazil; 6 Department of Tourism and Recreation, University of Life Sciences in Lublin, 15 Akademicka Str., 20-950 Lublin, Poland; 7 Department of Pharmacognosy with Medicinal Plants Garden, Medical University of Lublin, 1 Chodźki Str., 20-093 Lublin, Poland; 8 Department of Analysis and Food Quality Assessment, University of Life Sciences in Lublin, 8 Skromna Str., 20-704 Lublin, Poland

**Keywords:** cancer pain, natural products, monoterpenes, inclusion complex

## Abstract

Pain is one of the most prevalent and difficult to manage symptoms in cancer patients, and conventional drugs present a range of adverse reactions. The development of β-cyclodextrins (β-CD) complexes has been used to avoid physicochemical and pharmacological limitations due to the lipophilicity of compounds such as p-Cymene (PC), a monoterpene with antinociceptive effects. Our aim was to obtain, characterize, and measure the effect of the complex of p-cymene and β-cyclodextrin (PC/β-CD) in a cancer pain model. Initially, molecular docking was performed to predict the viability of complex formation. Afterward, PC/β-CD was obtained by slurry complexation, characterized by HPLC and NMR. Finally, PC/β-CD was tested in a Sarcoma 180 (S180)-induced pain model. Molecular docking indicated that the occurrence of interaction between PC and β-CD is favorable. PC/β-CD showed complexation efficiency of 82.61%, and NMR demonstrated PC complexation in the β-CD cavity. In the S180 cancer pain model, PC/β-CD significantly reduced the mechanical hyperalgesia, spontaneous nociception, and nociception induced by non-noxious palpation at the doses tested (*p* < 0.05) when compared to vehicle differently from free PC (*p* > 0.05). Therefore, the complexation of PC in β-CD was shown to improve the pharmacological effect of the drug as well as reducing the required dose.

## 1. Introduction

Among all the physical, psychological, and social effects and symptoms of cancer, pain is considered to be one of the most significant [1] due to its high prevalence, recently estimated at 44.5%, and its impact on the quality of life of patients [2,3].

Non-relieved pain is a significant public health problem [4]. The current main treatment for cancer pain is opioids (e.g., morphine and tramadol), but they present a range of adverse effects, such as constipation, nausea, and central nervous system problems. Patients can develop high tolerance, dependence, or active opioid use disorder, which limits the dosage and may cause early discontinuation of treatment and inadequate analgesia [5,6].

**Figure 1:**
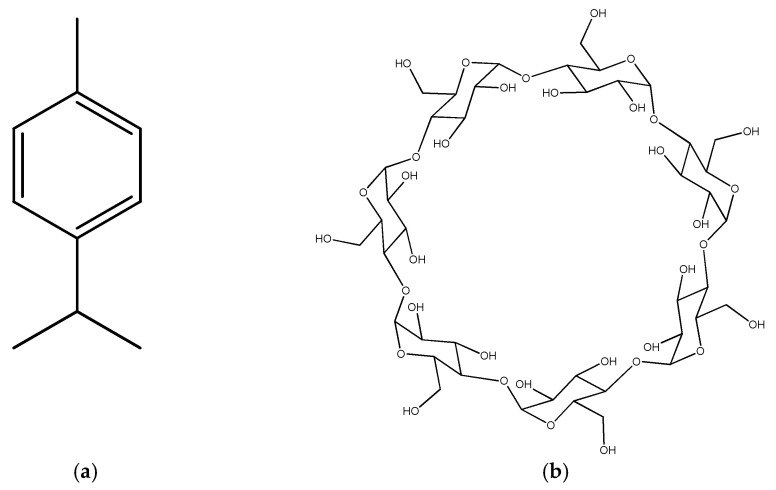
Chemical structures of p-cymene (**a**) and β-cyclodextrin (**b**).

The need for new analgesic drugs is, therefore, an important challenge, and the main target of this search are compounds from plants. One of the most promising of these are monoterpenes, organic compounds with great structural variety found in nature and present in several essential oils [7]. They have several biological properties, including analgesic properties [8,9].

p-Cymene (1-methyl-4-(1-methylethyl)-benzene; Figure 1) is a monoterpene hydrocarbon found in more than 100 plant species and commonly used for food or medical purposes, such as *Thymus vulgaris* (thyme) and *Origanum vulgare* L., as compiled by Marchese et al., 2017 [10]. Several studies have reported the anti-inflammatory and analgesic effects of p-cymene [11,12,13,14,15,16]. Recently, a study by our research group demonstrated the effect of p-cymene on cancer pain with activation of descending pain control pathways and modulation of calcium currents [17]. However, these compounds have low water solubility and bioavailability, limiting their use in inflammatory and painful conditions [18].

Several types of drugs with these same characteristics and properties have already been incorporated into drug delivery systems, such as cyclodextrins (CDs), which have the ability to form an inclusion complex with the guest molecule, improving its solubility, bioavailability, chemical stability and pharmacological effects [19,20]. Of the naturally occurring cyclodextrins, β-cyclodextrin (β-CD, Figure 1) consisting of seven glucopyranose units is most commonly used in pharmaceutical formulations as an excipient because of its efficiency in the complexation of aromatic molecules [21,22] and the ease of its production, with a subsequent low price compared to the others subtypes [23]. Moreover, β-CD is inert and has very low acute oral toxicity, presenting an oral median lethal dose (LD_50_) of greater than 3000 mg/kg for different experimental animals [24].

For these reasons, in the present study, we used a p-cymene/β-cyclodextrin (PC/β-CD) complex and isolated p-cymene (PC) to evaluate its antinociceptive activity in a cancer pain model using Sarcoma 180 (S180) cells and assessed the effectiveness of the complex in improving the pharmacological properties of this monoterpene.

## 2. Results

### 2.1. Docking Studies

**Figure 2:**
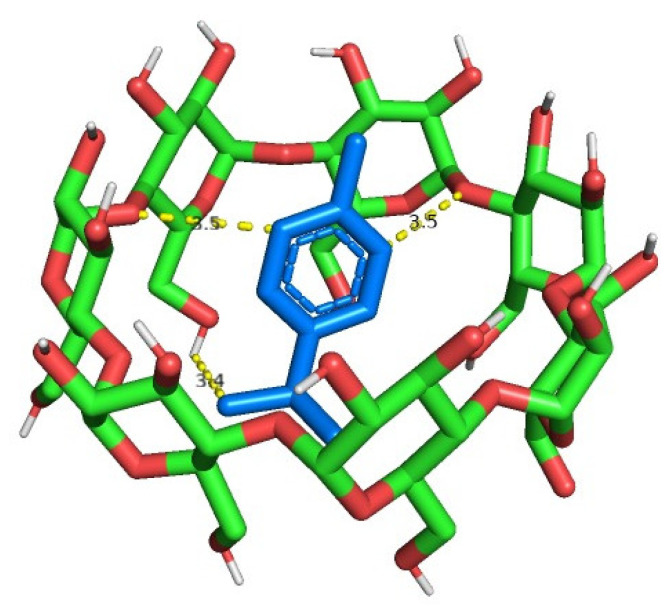
Representation of the binding mode of p-cymene (PC) in beta-cyclodextrin (β-CD). Blue represents the PC molecule, green represents the carbon atoms, and red represents the oxygen atoms. The dipole-induced dipole interactions are represented as dashed lines, and the values indicate the distances in Angstrom (Å).

In order to verify the existence of binding between PC and β-CD, a molecular docking analysis was performed. Figure 2 shows the ligand map of the best conformation from the binding complex PC/β-CD. It was possible to verify the presence of three dipole-induced dipole interactions, in which carbon atoms of p-cymene connect with two oxygen heteroatoms and one hydroxyl hydrogen of β-CD. We found a negative energy value of binding affinity of −3.9 kcal/mol.

### 2.2. Complexation Efficiency (CE%)

CE is a quantitative parameter to determine the amount of PC entrapped in the inclusion complex and was calculated using Equation (1), after determination of the linearity curve of the p-cymene standard at a concentration range (5–100 µg/mL) and at 210 nm (Figure S1). The physical mixture (PM) method obtained a CE of 0.77%, and the slurry complexation (SC) method presented a CE of 82.61%, showing that SC was the most efficient method for the inclusion of p-cymene in the β-CD cavity.

### 2.3. NMR Analysis

**Figure 3:**
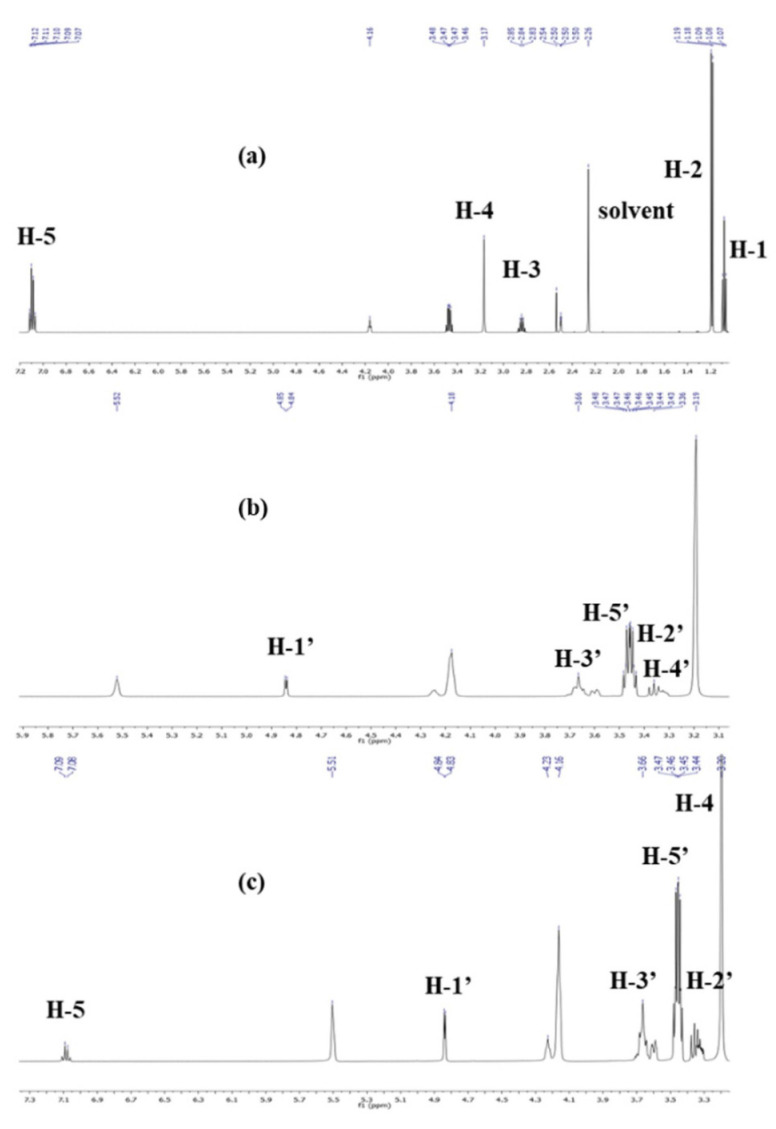
^1^H NMR spectra of free PC (**a**), β-CD (**b**), and the SC inclusion complex (**c**) in DMSO-d_6_.

Free PC, β-CD, and the SC inclusion complex were analyzed with 1H NMR spectroscopy. As shown in Figure 3, the complex showed the characteristic peaks of protons of both β-CD and PC. The NMR spectra of PC showed the characteristic peaks of H-1 protons at δ 1.08, H-3 protons at δ 2.84, and H-5 protons at δ 7.10 ppm. After complexation with β-CD, these peaks appeared at δ 1.06, δ 2.82, and δ 7.08 ppm, respectively. As Table 1 shows, the results suggest that the H-1 (∆δ), H-3, and H-5 protons (∆δ) presented a higher shift compared to the H-2 and H-4 protons (∆δ).

### 2.4. Pharmacological Evaluation

After characterization, considering the CE of 82.61% for the PC/β-CD complex obtained from the SC method, it was observed that the theoretical doses of 12.5, 25, and 50 mg/kg of PC utilized in the experimental protocols represent in truth approximately 1.2, 2.4, and 4.8 mg/kg of PC, respectively. For this reason, from here on, the results are expressed with the real doses of the studied monoterpene.

**Figure 4:**
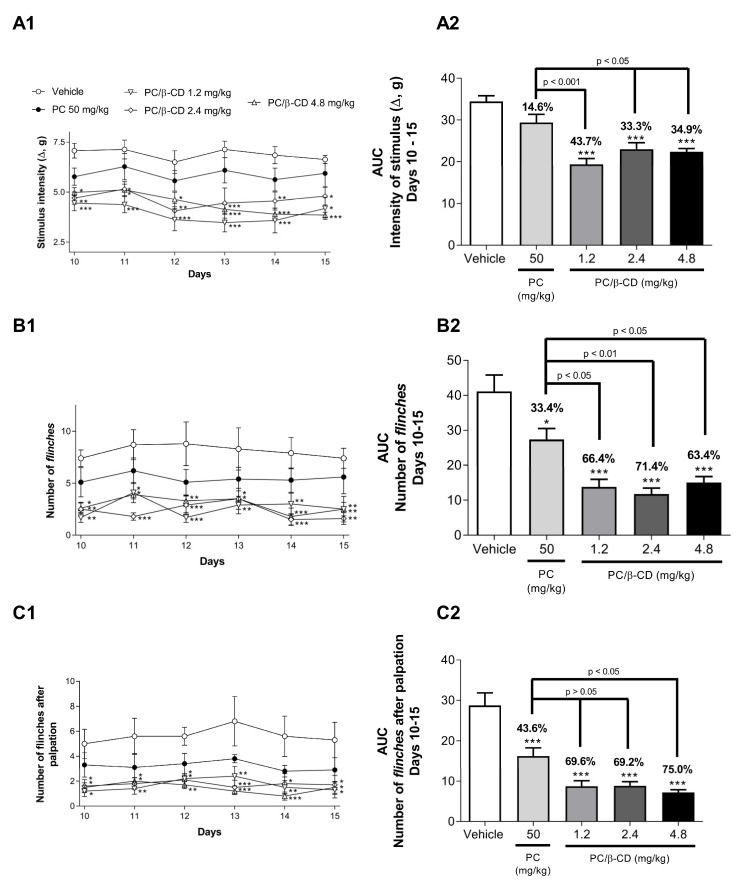
Effect of β-CD/PC complexes (1.2, 2.4, and 4.8 mg/kg, p.o.) and isolated PC (50 mg/kg, p.o.) on mechanical hyperalgesia ((**A1**): 10th to 15th day; (**A2**): areas under the curve from the 10th to the 15th day), spontaneous nociception ((**B1**): 10th to 15th day; (**B2**): areas under the curve from the 10th to the 15th day), and palpation ((**C1**): 10th to 15th day; (**C2**): areas under the curve from the 10th to the 15th day) induced by S180. The values are presented as mean ± S.E.M., * *p* < 0.05, ** *p* < 0.01, and *** *p* < 0.001 vs. vehicle group (two-way ANOVA followed by Tukey’s post hoc test).

PC/β-CD (1.2, 2.4, and 4.8 mg/kg) significantly reduced mechanical hyperalgesia from the 10th to 15th day when compared with animals treated with vehicle (Figure 4A1) and presented significant (*p* < 0.001 vs. vehicle, Figure 4A2) inhibition potential of 43.7, 33.3, and 34.9%, respectively. However, the free PC (50 mg/kg) administrated orally did not promote a significant reduction in hyperalgesia when compared to the vehicle group (*p* > 0.05).

By evaluating spontaneous nociception (Figure 4B1), all groups treated with the PC/β-CD complexes were able to significantly reduce the number of flinches presented compared with the vehicle group on all test days. Animals treated with PC/β-CD at all doses (1.2, 2.4, and 4.8 mg/kg) had significant inhibition potentials of 66.4, 71.4, and 63.4% (*p* < 0.001 vs. vehicle, Figure 4B2), respectively, when compared to the vehicle group. The group treated with free PC had no significant effect when compared to the vehicle group, and when compared to the PC/β-CD groups, there were significant differences (*p* < 0.05 PC/β-CD 1.2 and 2.4 mg/kg, *p* < 0.01 PC/β-CD 4.8 mg/kg), representing the greater effect of the complexes when compared to the free PC.

Later, we investigated the number of flinches presented by the animals after non-harmful palpation (Figure 4C1); it was found that all groups treated with the complex (1.2, 2.4, and 4.8 mg/kg) showed a significant difference (*p* < 0.001 vs. vehicle, Figure 4C2) when compared to the vehicle group, with inhibition potentials of 69.6, 69.2, and 75.0%, respectively. Once again, the free PC group did not obtain significant results when compared to the vehicle group (*p* > 0.05), whereas when compared to the PC/β-CD 4.8 mg/kg group, a significant difference was observed (*p* < 0.05).

Finally, the effect of all treatments on the use of the tumor-affected limb was tested, and no significant change was observed between all groups throughout the time course analyzed, including between the isolated PC and PC/β-CD groups (Figure S2).

Considering that β-cyclodextrin is an inert substance with very low acute oral toxicity [24], we sought to assess the acute toxicity of free p-cymene. Animals treated with PC (55, 175, and 550 mg/kg) were observed for 14 days, and no deaths and no behavioral changes indicative of toxicity were observed. A small reduction in body weight could be observed in mice of both sexes treated with a dose of 55 mg/kg and in males treated with 175 mg/kg. There was an increase in water consumption among males treated with doses of 55 and 175 mg/kg. In addition, an increase in feed intake was observed among females treated with 175 mg/kg, but this did not reflect an increase in the weight of the animals. From the biochemical analyses, it was possible to observe isolated alterations in the levels of alanine aminotransferase (ALT: males treated with 55 mg/kg), alkaline phosphatase (ALP), and creatinine (males treated with 550 mg/kg), and glucose (females treated with 175 mg/kg). No hematological changes were observed. In addition, no changes were found in the necropsied organs nor changes in their relative weight (Supplementary Material: Tables S1–S3).

## 3. Discussion

In this study, we demonstrate for the first time the molecular interactions established between p-cymene and β-CD, whose complexation demonstrated high efficiency through the SC method. Furthermore, we proved the benefits of complexation on the pharmacological response of this monoterpene in a cancer pain model.

Molecular docking analysis measures the spontaneity of binding between two molecules and binding energies with more negative values, indicating bigger interaction force [25]. In our study, we verified that the interaction between the PC and the β-CD is favorable (−3.9 kcal/mol). Similar to a previous study, in which the monoterpene citronellal complexed with β-CD obtained an energy value of −4.1 kcal/mol [26], indicating the ability of β-CD to encapsulate monoterpenes in its cavity to form complexes.

In fact, we observed that the inclusion complex obtained by the SC method presented high complexation efficiency (82.61%). This can be attributed to the procedure used in this method, the chemical–physical properties of p-cymene and β-CD, and the type of interaction between them [27]. Menezes et al. (2016) [28] obtained an complexation efficiency of 71.68% for carvacrol (biosynthetic derivative of PC) in β-CD (1:1 ratio) prepared by the SC method, which was lower than the result reported in this study. Our results show that SC is an efficient method of producing the PC/β-CD inclusion complex, which corroborates other published results by Serafini et al. (2012) [29].

Additionally, NMR analysis is commonly used to study the formation and the interaction of the inclusion complex of β-CD and guest molecules [30]. Due to the presence of the aromatic ring, the inclusion of aromatic compounds typically has a pronounced effect on the chemical shifts of protons in the β-CD cavity. In the present study, the formation of the inclusion complex by insertion of the aromatic ring of PC into the β-CD lipophilic cavity was demonstrated by the chemical shifts of the H-3 and H-5 resonances of the β-CD. These protons are present in the inner surface of β-CD and interact more with PC molecules present in its cavity than the H-1 protons that are on the outer surface of the β-CD cavity. This result indicated that the PC molecules were located in the β-CD cavity.

Several benefits of cyclodextrins such as improved bioavailability and solubility, among others, have already been studied (Tiwari et al. 2010). However, a patent review conducted by De Oliveira et al., (2015) [31] reported that the use of cyclodextrins in therapies for painful conditions allows the development of new treatments with a reduction in the dosage of analgesic drugs, as well as a reduction in their side effects. In fact, in our study we verified that, after characterization and considering the CE of 82.61% of the PC/β-CD complex, the theoretical doses of PC (12.5, 25, and 50 mg/kg utilized in the experimental protocols) represented 1.2, 2.4, and 4.8 mg/kg of PC, respectively. These theoretical doses were initially established based on a previous study of PC on cancer pain [17], but only after the characterization was it possible to establish the correct doses for the monoterpene under study.

Then, our study shows the effect of PC alone and complexed in β-CD from the tenth day of a mouse model, the period in which the animals begin to present hyperalgesic behavior due to the time the tumor takes to develop [17,32,33]. The analysis continued until the 15th day; after this period, it would be impossible to continue due to the exacerbated growth of the tumor in the paw of the mice. In order to evaluate the different modalities of pain experienced by patients with cancer, some behavioral tests have been conducted to mimic what often happens in clinical practice [34,35].

In fact, neurochemical alterations can produce many kinds of pain related to cancer, such as hyperalgesia, spontaneous nociception, and allodynia, which are associated with alterations in the threshold of sensitization of specific nociceptors (fibers A-δ, Cm and A-β, respectively) [35]. The present results corroborate previous studies that demonstrated an improvement in the antinociceptive effect of PC when complexed with β-CD, as well as a longer duration of effect in acute pain protocols [18].

Unlike the PC/β-CD complex, in this study, free PC (50 mg/kg) when administered by the oral route did not present any effect in the behavioral tests applied, differing from a previous study that used free PC administered by a subcutaneous route in which it reduced nociceptive responses [17]. Hu et al. (2015) [36] showed that p-cymene is rapidly absorbed after oral administration (40 mg/kg) in rats, presenting a time of maximum concentration of 0.33 ± 0.11 h and a half-life of 0.44 ± 0.07 h, which demonstrates the rapid absorption and elimination of this monoterpene. In fact, this compound is oxidized mainly to cuminyl alcohol and cumic acid, among others [37,38]. For these metabolites, the analgesic effect of only cuminyl alcohol (6.5 to 400 mg/kg, i.p.) is known in acute and chronic models, possibly involving opioid receptors, the L-arginine/NO/cGMP pathway, and anti-inflammatory functions [39]. These previously published pharmacokinetic data help us to suppose that, in our study, no effect of free PC was verified due to the low dose administered orally (50 mg/kg), with the compound under study being susceptible to the hepatic and renal mechanisms of elimination. In addition, behavioral assessments were performed after 60 min, at which time the plasma concentration of p-cymene had already significantly reduced, considering T1/2 = 0.44 h, as demonstrated by Hu et al. (2015) [36].

However, the PC/β-CD complex showed analgesic effect at all doses. In fact, the complexation of substances in CDs is known to improve the in vivo stability against hydrolysis, oxidation, decomposition, and dehydration, along with a bioavailability increase in the guest molecule. A review conducted by Lima et al. (2016) [40] about the interaction between terpenes and CDs reported that some studies demonstrated an improvement in the absorption of terpenes and an increase in their pharmacological efficacy when administered by the oral route carried in inclusion complexes. However, the studies lacked information about how the complex formation improved these aspects.

Even though the oral route is the most convenient and accepted route for the administration of medications, the physicochemical properties of the drugs must be specific for this method of administration and not suffer from the first-pass effect, which reduces the concentration of a drug before it reaches the systemic circulation, thereby leading to poor bioavailability [41]. Compounds from medicinal plants, such as terpenes, are known for their low water solubility and poor bioavailability [40]. On the other hand, a formation of a complex between a drug and cyclodextrin is established in the literature as a process that improves drug solubility, consequently improving oral bioavailability [42].

**Figure 5:**
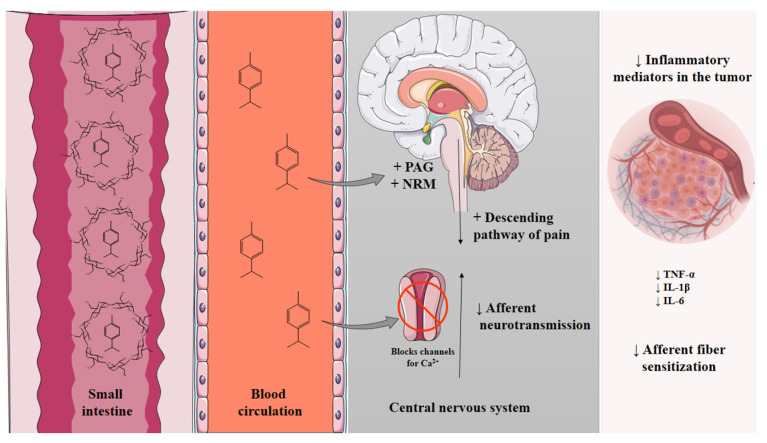
Mechanism of action of the β-CD/PC complex against cancer pain.

In respect to how PC exerts its antinociceptive effect (Figure 5), it has been shown that it acts through some known mechanisms, such as the modulation of leukocyte migration, leading to decreased release of cytokines (TNF-α, IL-1β, and IL-6), as well as a reduction in prostanoids and sympathomimetic amines (dopamine) [13]. These substances are present in the tumor microenvironment and are responsible for sensitizing the sensory terminals of A-δ, A-β, and C fibers, generating painful stimuli.

Moreover, a previous study by our group demonstrated that PC causes alterations in Fos protein expression, an important marker of neuronal activity, inducing activation of regions involved in the descending pain inhibitory pathways, such as the periaqueductal gray (PAG) and nucleus raphe magnus (NRM) [17]. In addition, PC has been shown to have an effect on voltage-dependent calcium channel current modulation, a well-established mechanism for mediating pain signals in primary afferent neurons [17].

Other studies show that PC may act on both peripheral and central nervous system pathways, probably by modulation of opioid receptors and cytokine release [12,13]. Additionally, Quintans et al. (2013) [18] demonstrated that the complexation of PC in β-CD significantly improved the effects of the compound in an animal model of pain and inflammation.

Thus, considering the mechanisms of action already studied for p-cymene and the effects of complexation on the pharmacokinetics of the host compound, we can propose that the β-CD/PC complex can act as shown in the illustration below (Figure 5). After oral administration of the complex, intestinal bacteria and enzymes hydrolyze the β-CD structure [24], releasing the PC that is absorbed. At this point, it is observed that complexation in β-CD protects the drug molecule, increasing its stability, with a consequent increase in bioavailability, as already demonstrated in the literature [43,44]. After, PC accesses its sites of action through the bloodstream. Thus, this compound can activate the pain inhibitory descending pathway, modulate calcium channels and neurotransmission, in addition to acting peripherally, reducing the production of inflammatory mediators that increase the sensitization of the regions affected by the tumor [15,16,17,18].

As for the acute toxicity of p-cymene, some isolated changes were observed between the sexes, such as a reduction in body weight, but this decrease was not associated with a reduction in food intake. On the other hand, the increase in feed intake among females treated with the 175 mg/kg dose may explain the increase in blood glucose levels in this experimental group. As for the other biochemical parameters, there was an increase in ALT (males treated with 55 mg/kg), creatinine, and alkaline phosphatase (males treated with 550 mg/kg), indicating a possible alteration in renal and hepatic function in these animals. However, in the other groups, these parameters were within normal limits, and no alterations in the kidneys and livers of the animals were observed. Thus, these results indicate that free PC, at the doses tested, has low toxicity. In fact, p-cymene is recognized as “Generally Recognized as Safe” (GRAS) by the United States Food and Drug Administration and presents oral LD50 of 4750 mg/kg in rats [45]. It is noteworthy that the use of complexing this compound in β-cyclodextrin improves its pharmacological effects, requiring a dose to reduce cancer pain almost 11 times lower than the lowest dose used in the acute toxicity test, which further increases the safety of its use.

All these findings demonstrate the potential of p-cymene as an analgesic compound for the treatment of chronic painful conditions. However, our study presents as limitations the absence of a comparative pharmacokinetic analysis between the developed complex and the free compound as well as the safety profile (subacute toxicity) of the compound used. We hope that future studies can fill this knowledge gap and contribute to better elucidating the benefits of complexation in the pharmacological effect of p-cymene.

## 4. Materials and Methods

### 4.1. Docking Studies

Initially, molecular docking was performed to calculate the binding of the p-cymene and beta-cyclodextrin in the complex, in order to predict the viability of complex formation between PC and β-CD. The 3D structure of β-CD and PC was extracted from the protein complex of the β-CD/maltodextrin binding protein (Protein Data Bank [PDB] code: 1DMB) and complex Ru(p-cymene)/apo-Fr (PDB code: 3O7S), respectively. Openbabel was used to convert the molecules’ structure files to PDBQT format [42]. Docking analysis was then performed using AutodockVina [43], implemented using the program Pyrx [44]. All the parameters were selected as default. The interactions between the PC and β-CD were evaluated using the PyMOL Molecular Graphics System (Version 2.2, Schrödinger, LLC., New York, NY, USA), selecting the conformation with the best binding affinity.

### 4.2. Obtaining and Characterizing the PC/β-CD Complex

#### 4.2.1. Drugs and Chemicals

The test compounds used in this study were p-cymene (PC) (1-methyl-4-(1-methylethyl)-benzene, 99.7% purity), β-cyclodextrin (β-CD, ≥97% purity), cremophor, and sodium chloride, which were obtained from Sigma Aldrich (St. Louis, MO, USA); morphine and lactated Ringer’s solution were purchased from Cristália (São Paulo, Brazil).

#### 4.2.2. Preparation of the Complexes

Slurry complexation (SC) was carried out by the addition of water to a beaker containing 3.405 g of β-CD (3:4, *v*/*w*). Approximately 402.6 mg of PC, which is equal to about a 1:1 molar guest:host ratio, was added to the slurry and stirred for 40 min by a magnetic stirring device operating at 400 rpm (Quimis Q 261A21, São Paulo, Brazil). The mixture was then heated to 34 °C for 2 h in the same device, transferred to an agate mortar, and dried in a desiccator. In order to compare the efficiency of the SC method, a physical mixture (PM) was prepared by the addition of PC to an agate mortar containing powdered β-CD under manual agitation. The PC/β-CD 1:1 molar ratio was maintained as described for the inclusion complex preparation, and the mechanical mixture was stored in airtight glass containers [28].

#### 4.2.3. Complexation Efficiency (CE%)

The inclusion efficiency of p-cymene in the inclusion complex was determined by the ultrasonic–centrifugal method [45]. The amount of PC in the inclusion complex was analyzed by HPLC. The calibration curve of the PC concentration was y = 84,052x + 29,422 (R^2^ = 0.999). All preparations and measurements were made in triplicate. The CE was calculated according to Equation (1):

CE% = [p-cymene experimental (mg)/p-cymene theoretical (mg)] × 100(1)

where “p-cymene experimental” is the quantified amount of p-cymene in the solid inclusion complex and “p-cymene theoretical” is the amount of p-cymene initially used to prepare the inclusion complex.

#### 4.2.4. Nuclear Magnetic Resonance (NMR)

The ^1^H NMR spectra of p-cymene, β-CD, and the SC inclusion complex were recorded with a Varian VNMRS 500 MHz spectrometer. The samples were dissolved in DMSO-d_6_ at 298 K in 5 mm tubes. Chemical shifts were shown in ppm with DMSO-d6 (2.50 ppm) as the internal standard.

### 4.3. Pharmacological Evaluation

#### 4.3.1. Animals

Male Swiss mice (between 30–40 g; 2–3 months of age) obtained from the Animal Facilities of the Federal University of Sergipe were used in the experiments. All handling procedures and experimental protocols were approved by the Animal Care and Use Committee (CEPA/UFS 20/2019; 71/2010) and followed the recommendations of the NRC Guide for the Care and Use of Laboratory Animals and the International Association for the Study of Pain (IASP) for the use of animals in pain research [46]. The animals were housed randomly in appropriate cages at 21 ± 2 °C with a 12 h light/dark cycle with free access to food (Purina^®^, Vargeão, Brazil) and water.

#### 4.3.2. Tumor Cell and Implantation

Sarcoma 180 (S180) tumor cells were obtained from the Laboratory of Experimental Oncology at the Federal University of Ceará and inoculated in the intraperitoneal region of a maintenance animal according to previous protocols [32,33,47].

#### 4.3.3. Treatment and Behavioral Studies

Twenty-four hours after S180 administration, the animals (n = 10/group) were treated by the oral route (p.o.) for 15 consecutive days with vehicle (saline + Cremophor 0.4% *v*/*v*), PC (50 mg/kg), or the PC/β-CD complex obtained by the SC method. Initially, the theoretical doses of the PC/β-CD complex were 12.5, 25, and 50 mg/kg, but after characterization, they represented 1.2, 2.4, and 4.8 mg/kg of PC, respectively. From the ninth day of the treatment, behavioral analyses were conducted on alternating days, 60 min after treatment. The observer responsible for the evaluation was blinded to the experimental situation of each animal.

#### 4.3.4. Mechanical Hyperalgesia

To assess the mechanical sensitivity of the treated mice, the Von Frey’s test was used (electronic aesthesiometer, EFF-301 model, Insight^®^, Ribeirão Preto, Brazil) in the paw containing the tumor as previously described [33].

#### 4.3.5. Spontaneous and Palpation-Induced Nociception

The animals were placed in mirrored boxes and allowed to acclimate for 10 min and then evaluated for spontaneous pain and then for non-noxious palpation nociception, following previous protocols [48].

#### 4.3.6. Movement-Evoked Pain

Limb use was evaluated through observation of the mice while they walked in the mirrored boxes, as described by King et al., 2007. The behavior of limping and/or guarding of the hind limb containing the sarcoma cells was rated using the following scale: 0 = absence of use, 1 = barely used, 2 = limp and guard, 3 = limp, and 4 = normal walk [33].

#### 4.3.7. Acute Oral Toxicity

The acute toxicity of free p-cymene was determined according to Organization for Economic Cooperation and Development (OECD) guidelines [49]. Swiss mice (n = 05/both genders) were used, which were treated with PC at doses of 55, 175, and 550 mg/kg (p.o.). The control group received the vehicle (saline + cremophor). The behavioral parameters of toxicity, weight evolution, and water and food consumption were evaluated for 14 days. On the 15th day, the animals were anesthetized by administration of ketamine (1 g/mL, 0.1 mL/kg body weight, i.p.) and xylazine (2 g/mL, 0.1 mL/kg, i.p.), and their blood (4 mL) was collected through the superior mesenteric artery. After, 1 mL was added to a tube containing EDTA (ethylenediaminetetraacetic acid), to perform hematological analyses in an automated analyzer (BC5380, Mindray). Moreover, 3 mL of blood was added to a dry tube and centrifuged, and the serum was removed for later biochemical analysis in an automated analyzer (Abbott; Architect C 8000), with kits from Clinica Chemistry^®^. Finally, some organs such as the liver, heart, brain, lungs, kidneys, and spleen were weighed and autopsied. Their relative weight was calculated ((organ weight/body weight) × 100).

#### 4.3.8. Statistical Analysis

The data obtained were submitted to a Student’s *t*-test for comparison of two means, and for more than two means, one-way or two-way analyses of variance (ANOVA) were used with the results expressed by the mean ± standard error of the mean (SEM), followed by Tukey’s test. The differences were considered significant if *p* < 0.05. Graph Pad Prism (v 8.00) software (San Diego, CA, USA) was used. The percentage of inhibition was determined using the following formula: inhibition% (PI) = 100 * (control − experiment)/control, with the data obtained by the area under the curve (AUC).

## 5. Conclusions

Thus, our results clearly demonstrate an improvement in the effect of PC when complexed with β-CD by the SC method. The characterization of the complex was proven by NMR and docking, showing the possibility of using this technology in the physicochemical and pharmacological improvement of monoterpene compounds. However, pharmacokinetic studies are needed to more accurately measure the impact of complexation on the bioavailability and pharmacological effect of the studied monoterpene.

## Figures and Tables

**Table 1:** Complexation efficiency (CE) values determined for PM and SC PC/β-CD inclusion complexes using HPLC at 210 nm.

PC/β-CD Samples	CE (%) *
Physical mixture (PM)	0.77 ^a^ ± 0.82
Slurry complexation (SC)	82.61 ^b^ ± 0.58

* The values given are averages of three replicate samples; standard deviations are displayed. CE values with differing superscript letters indicate significantly different values (*p* < 0.05, Student’s *t*-test).

## Data Availability

Data generated or analyzed during this study are provided in full within the published article.

## Supplementary Materials

The following supporting information can be downloaded at: https://www.mdpi.com/article/10.3390/molecules28114465/s1. Figure S1. Linearity curve of the p-cymene standard at a concentration range of (5–100 µg/mL) and at 210 nm (A), and signal response (8.49 min) of p-cymene at a 100 µg/mL concentration (B). Figure S2. Effect of PC/β-CD complexes (1.2, 2.4, and 4.8 mg/kg, p.o.) and isolated PC (50 mg/kg, p.o.) on paw use in animals with an S180 tumor. The values are expressed as the mean ± S.E.M. Table S1: Body weight, water e and feed consumption of male and female mice (n = 05/group) submitted to acute treatment with PC (55, 175 or 550 mg/kg, p.o.). The values are expressed as Mean ± E.P.M. of the parameters evaluated during 14 days. ANOVA follow Tukey’s test: * *p* < 0.05 vs vehicle; ** *p* < 0.01 vs vehicle. Table S2: Relative (Absolute organ weight * 100/Animal final weight) organ weight of mice at the end of acute dosing of PC at different doses. Values are expressed as mean ± S.E.M. no significant difference was identified. Table S3: Biochemical parameters of male and female mice ate the end of acute toxicity study. Values are expressed as mean ± S.E.M. ANOVA follow Tukey’s test: * *p* < 0.05 vs vehicle; ** *p* < 0.01 vs vehicle.

## Sample Availability

Samples of the compounds are available from the authors.
