# Supplementary material for: Antinociceptive Effect of a p-Cymene/β-Cyclodextrin Inclusion Complex in a Murine Cancer Pain Model: Characterization Aided through a Docking Study
Source: Molecules. 2023 May 31;28(11):4465. doi: 10.3390/molecules28114465 (PMC10254339; doi:10.3390/molecules28114465)
Supplement: Supplementary file 1 [file molecules-28-04465-s001.zip › molecules-2352685-supplementary.pdf]

Supplementary information

HPLC method for quantification of p-cymene into β-CD

The *p*-cymene content in the samples was determined with an HPLC instrument (model Shimadzu®) consisted of a vaccum degasser (model DGU-20A3), a binary pump (model LC-20 AD), an autosampler (model SIL-20A) and a diode array detector (model SPD-M20A vp) with interface CBM-20A. A reverse phase separation chromatography was achieved on the Phenomenex C_{18} analytical column (150 mm x 4.6 mm i.d., 5 µm). The isocratic mobile phase was a combination of methanol/acetonitrile (MeCN): water (H_2O) (80:20) for 15 min run time with 1.0 mL min^{-1} flow rate. Injection volume for all samples and standard solutions was 20 µL and analysis was performed at ambient temperature.

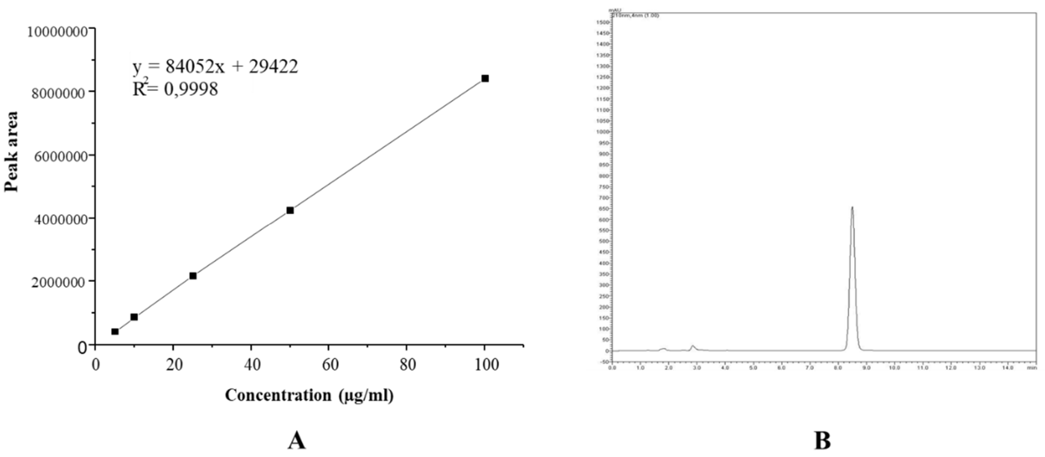

Figure S1. Linearity curve of *p*-cymene standard at concentration range (5-100 µg/ml) at 210 nm (A) and signal response (8.49 min) of *p*-cymene at 100 µg/ml concentration (B).

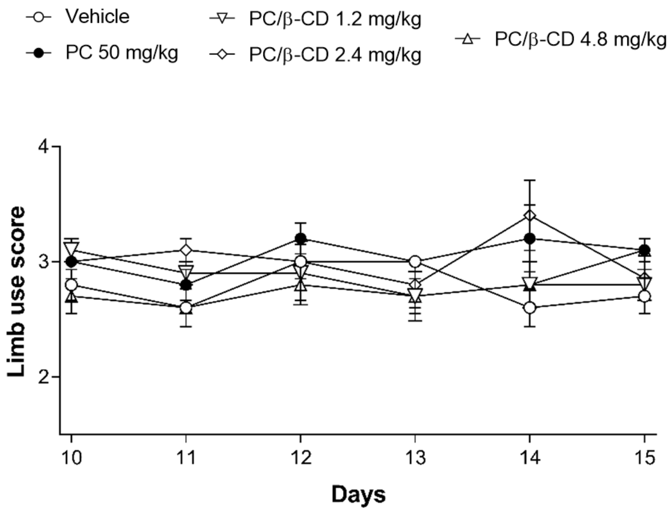

Figure S2. Effect of PC/β-CD complexes (1.2, 2.4 and 4.8 mg/kg, p.o.) and isolated PC (50 mg/kg, p.o.) on paw use in animals with S180 tumor. Values are expressed as mean ± S.E.M.

Table S1: Body weight, water e and feed consumption of male and female mice (n = 05/group) submitted to acute treatment with PC (55, 175 or 550 mg/kg, p.o.). The values are expressed as Mean ± E.P.M. of the parameters evaluated during 14 days. ANOVA follow Tukey's test: *p < 0.05 *vs* vehicle; **p < 0.01 *vs* vehicle.

Treatment	Body weight (g)		Water (ml)		Food (g)	
	Male	Female	Male	Female	Male	Female
**Vehicle**	32.4 ± 0.5	26.4 ± 0.3	13.5 ± 1.3	8.9 ± 0.9	9.5 ± 0.8	6.4 ± 0.4
**PC 55 mg/kg**	28.3 ± 0.9**	23.7 ± 0.4**	23.1 ± 2.8*	10.2 ± 1.2	9.1 ± 1.1	6.8 ± 0.2
**PC 175 mg/kg**	29.6 ± 0.5*	27.2 ± 0.6	23.3 ± 2.4*	12.6 ± 1.2	10.5 ± 1.6	8.8 ± 0.4*
**PC 550 mg/kg**	33.6 ± 0.4	26.8 ± 0.4	17.5 ± 1.3	13.9 ± 1.7	10.9 ± 0.9	7.5 ± 0.8

Table S2: Relative (Absolute organ weight * 100/Animal final weight) organ weight of mice at the end of acute dosing of PC at different doses. Values are expressed as mean ± S.E.M. no significant difference was identified.

	Relative weight of organs (%)						
	**Liver**	**Spleen**	**Kidney**	**Brain**	**Lung**	**Heart**	**Stomach**
**Vehicle ♂**	5.0 ± 0.2	0.7 ± 0.1	1.6 ± 0.0	1.3 ± 0.1	0.8 ± 0.0	0.5 ± 0.0	1.6 ± 0.2
**PC 55 mg/kg ♂**	5.2 ± 0.1	1.3 ± 0.1	1.7 ± 0.1	1.4 ± 0.1	0.7 ± 0.1	0.6 ± 0.0	0.8 ± 0.1
**PC 175 mg/kg ♂**	4.9 ± 0.4	0.9 ± 0.2	1.6 ± 0.0	1.1 ± 0.2	0.7 ± 0.1	0.8 ± 0.3	0.8 ± 0.4
**PC 550 mg/kg ♂**	5.4 ± 0.3	0.8 ± 0.1	1.5 ± 0.0	1.2 ± 0.1	0.7 ± 0.1	0.5 ± 0.0	1.3 ± 0.1
**Vehicle ♀**	5.0 ± 0.3	0.7 ± 0.0	1.5 ± 0.0	1.4 ± 0.0	1.0 ± 0.1	0.5 ± 0.0	1.5 ± 0.1
**PC 55 mg/kg ♀**	4.9 ± 0.4	0.7 ± 0.1	1.7 ± 0.1	1.3 ± 0.2	1.6 ± 0.6	0.9 ± 0.4	1.4 ± 0.1
**PC 175 mg/kg ♀**	5.2 ± 0.2	0.9 ± 0.3	1.6 ± 0.1	1.4 ± 0.2	0.8 ± 0.1	0.5 ± 0.0	1.5 ± 0.2
**PC 550 mg/kg ♀**	5.2 ± 0.3	0.6 ± 0.1	1.5 ± 0.1	1.5 ± 0.0	1.1 ± 0.2	0.4 ± 0.5	1.3 ± 0.1

Table S3: Biochemical parameters of male and female mice ate the end of acute toxicity study. Values are expressed as mean ± S.E.M. ANOVA follow Tukey's test: *p < 0.05 *vs* vehicle; **p < 0.01 *vs* vehicle.

Biochemical parameters	**Male**				**Female**			
	**Vehicle**	**PC 55 mg/kg**	**PC 175 mg/kg**	**PC 550 mg/kg**	**Vehicle**	**PC 55 mg/kg**	**PC 175 mg/kg**	**PC 550 mg/kg**
Urea (mg/dL)	39.5 ± 4.1	60.8 ± 9.4	65.8 ± 12.1	42.5 ± 5.1	36.5 ± 3.9	44.6 ± 7.7	36.2 ± 3.4	32.6 ± 8.2
Uric acid (mg/dL)	2.9 ± 0.1	3.2 ± 0.4	3.2 ± 0.1	3.6 ± 0.2	2.9 ± 0.2	3.4 ± 0.6	3.8 ± 0.1	3.2 ± 0.1
Creatinine (mg/dL)	0.2 ± 0.0	0.3 ± 0.0	0.3 ± 0.0	*0.4 ± 0.0***	0.3 ± 0.0	0.4 ± 0.0	0.4 ± 0.0	0.4 ± 0.0
Total protein (mg/dL)	5.5 ± 0.1	4.7 ± 0.3	5.0 ± 0.4	6.5 ± 0.1	5.9 ± 0.1	6.1 ± 0.1	6.5 ± 0.3	6.8 ± 0.5
Albumin (mg/dL)	2.3 ± 0.0	2.2 ± 0.1	2.3 ± 0.0	2.5 ± 0.1	2.6 ± 0.0	2.6 ± 0.0	2.7 ± 0.1	2.5 ± 0.1
Globulin (mg/dL)	3.2 ± 0.14	2.3 ± 0.3	2.7 ± 0.4	3.9 ± 0.1	3.4 ± 0.2	3.3 ± 0.0	3.3 ± 0.7	4.3 ± 0.5
AST (U/L)	125.0 ± 24.9	178.0 ± 40.8	129.3 ± 23.1	96.6 ± 5.1	160.6 ± 27.4	114.0 ± 11.8	139.4 ± 41.0	99.6 ± 8.5
ALT (U/L)	56.8 ± 5.0	102.6 ± 9.3**	69.0 ± 11.0	61.0 ± 6.8	60.6 ± 7.7	54.6 ± 3.7	103.0 ± 50.7	50.2 ± 4.1
ALP (U/L)	79.8 ± 7.4	106.1 ± 8.1	99.5 ± 7.8	114.6 ± 9.5*	195.9 ± 39.3	142.2 ± 19.8	126.8 ± 11.8	118.0 ± 2.5
Glucose (mg/dl)	28.6 ± 5.7	63.7 ± 17.3	43.8 ± 13.7	81.0 ± 10.7	55.7 ± 6.3	60.2 ± 15.6	101.4 ± 12.0*	80.2 ± 3.6
Hematological parameters	**Male**				**Female**			
	**Vehicle**	**PC 55 mg/kg**	**PC 175 mg/kg**	**PC 550 mg/kg**	**Vehicle**	**PC 55 mg/kg**	**PC 175 mg/kg**	**PC 550 mg/kg**
Red blood cells ($10^6/mm^3$)	10.6 ± 0.7	8.8 ± 0.5	9.4 ± 0.2	10.1 ± 0.3	9.8 ± 0.3	9.4 ± 0.5	9.6 ± 0.2	10.2 ± 0.3
Hemoglobin (g/dL)	17.0 ± 0.9	13.7 ± 1.1	14.9 ± 0.3	15.6 ± 0.4	15.4 ± 0.7	14.2 ± 0.9	15.0 ± 0.5	15.8 ± 0.4
Hematocrit (%)	49.1 ± 2.6	40.8 ± 3.0	44.3 ± 0.9	46.0 ± 1.1	45.2 ± 1.9	42.1 ± 2.3	44.6 ± 1.1	46.7 ± 1.1
Leukocytes ($10^6/mm^3$)	6.5 ± 1.0	7.6 ± 0.9	7.0 ± 1.0	6.7 ± 0.9	13.4 ± 3.3	7.6 ± 0.9	7.2 ± 1.1	7.2 ± 1.0
Neutrophils (%)	4.9 ± 0.5	6.6 ± 1.1	7.7 ± 1.8	7.0 ± 1.7	6.5 ± 0.7	7.0 ± 0.9	6.7 ± 1.7	5.3 ± 0.9
Lymphocytes (%)	91.5 ± 0.8	89.0 ± 0.9	87.9 ± 2.1	89.8 ± 1.7	90.2 ± 0.3	88.5 ± 0.9	86.6 ± 2.8	91.2 ± 1.0
Monocytes (%)	3.2 ± 0.5	3.9 ± 0.3	3.7 ± 0.4	3.0 ± 0.3	2.9 ± 0.4	4.1 ± 0.4	3.9 ± 0.6	3.1 ± 0.3
Eosinophils (%)	0.1 ± 0.0	0.1 ± 0.0	0.1 ± 0.0	0.1 ± 0.0	0.0 ± 0.0	0.1 ± 0.0	0.0 ± 0.0	0.1 ± 0.0

ALP – Alkaline phosphatase; AST - Aspartate aminotransferase; ALT - Alanine aminotransferase.
